# Molecular prognosticators in clinically and pathologically distinct cohorts of head and neck squamous cell carcinoma—A meta-analysis approach

**DOI:** 10.1371/journal.pone.0218989

**Published:** 2019-07-16

**Authors:** Ram Bhupal Reddy, Samanta S. Khora, Amritha Suresh

**Affiliations:** 1 Integrated Head and Neck Oncology Program, Mazumdar Shaw Centre for Translational Research, Mazumdar Shaw Medical Foundation, Narayana Health, Bangalore, Karnataka, India; 2 Head and Neck Oncology, Mazumdar Shaw Medical Centre, Narayana Health, Bangalore, Karnataka, India; 3 Division of Medical Biotechnology, School of Biosciences and Technology, Vellore Institute of Technology, Vellore, Tamil Nadu, India; University of Nebraska Medical Center, UNITED STATES

## Abstract

Head and neck squamous cell carcinomas (HNSCC) includes multiple subsites that exhibit differential treatment outcome, which is in turn reflective of tumor stage/histopathology and molecular profile. This study hypothesized that the molecular profile is an accurate prognostic adjunct in patients triaged based on clinico-pathological characteristics. Towards this effect, publically available micro-array datasets (n = 8), were downloaded, classified based on HPV association (n = 83) and site (tongue n = 88; laryngopharynx n = 53; oropharynx n = 51) and re-analyzed (Genespring; v13.1). The significant genes were validated in respective cohorts in The Cancer Genome Atlas (TCGA) for correlation with clinico-pathological parameters/survival. The gene entities (n = 3258) identified from HPV based analysis, when validated in TCGA identified the subset specifically altered in HPV+ HNSCC (n = 63), with three genes showing survival impact (RPP25, NUDCD2, NOVA1). Site-specific meta-analysis identified respective differentials (tongue: 3508, laryngopharynx: 4893, oropharynx: 2386); validation in TCGA revealed markers with high incidence (altered in >10% of patients) in tongue (n = 331), laryngopharynx (n = 701) and oropharynx (n = 404). Assessment of these genes in clinical sub-cohorts of TCGA indicated that early stage tongue (MTFR1, C8ORF33, OTUD6B) and laryngeal cancers (TWISTNB, KLHL13 and UBE2Q1) were defined by distinct prognosticators. Similarly, correlation with perineural/angiolymophatic invasion, identified discrete marker panels with survival impact (tongue: NUDCD1, PRKC1; laryngopharynx: SLC4A1AP, PIK3CA, AP2M1). Alterations in ANO1, NUDCD1, PIK3CA defined survival in tongue cancer patients with nodal metastasis (node+ECS-), while EPS8 is a significant differential in node+ECS- laryngopharyngeal cancers. In oropharynx, wherein HPV is a major etiological factor, distinct prognosticators were identified in HPV+ (ECHDC2, HERC5, GGT6) and HPV- (GRB10, EMILIN1, FNDC1). Meta-analysis in combination with TCGA validation carried out in this study emphasized on the molecular heterogeneity inherent within HNSCC; the feasibility of leveraging this information for improving prognostic efficacy is also established. Subject to large scale clinical validation, the marker panel identified in this study can prove to be valuable prognostic adjuncts.

## 1. Introduction

Accurate molecular prognosticators predictive of survival in patients diagnosed with cancer can be an invaluable adjunct to the existing clinical and pathological parameters. In head and neck squamous cell carcinoma (HNSCC), advanced stage tumors, nodal metastasis, and presence of aggressive pathological features (perineural (PNI)/lymphovascular invasion (LVI)) are poor prognosticators indicating an increased risk of metastatic disease and reduced disease free/overall survival [1–3]. Notwithstanding the adoption of multi-modal treatment strategies based on these parameters, survival has not improved [4], indicating the need for additional and more accurate factors to improve disease management. Currently, TNM staging is a primary parameter for triaging the patients based on prognosis, however stage-independent prognostic impact attributed to the presence of pathological parameters such as PNI, angiolymphatic invasion (ALI) and LVI [5,6] indicates the need for a multi-parameter assessment. Whether additional consideration of underlying molecular parameters can further improve prognostication needs to be investigated.

Prognostic impact of the clinico-pathological parameters is site-dependent; recent studies have identified differential impact of the pathological parameters in the prognosis of tongue, buccal and laryngopharyngeal cancers possibly owing to the underlying molecular pathways involved in the carcinogenesis and progression of the disease [7–9]. Additionally, Human papilloma virus (HPV) -associated etiology is an increasingly evident prognostic factor; oropharynx being the most commonly associated site [10] [11] [12]. Among pathological parameters, nodal metastasis is a significant prognosticator in HNSCC; the primary tumor site, presence of PNI, extra-capsular spread (ECS), being additional contributing factors [2–4,13,14]. PNI is a factor that can determine lymph node involvement, recurrence, disease-free and overall survival necessitating appropriate treatment management if susceptibility is detected. Elective neck dissection is prescribed for T1/T2N0 PNI+ patients [15,16]. Additionally, PNI was identified as a prognostic factor in early stage tongue cancer, while LVI was a significant parameter in late stage buccal mucosal cancers [3]. These studies point out to etiological/biology-driven differences in the survival impact of the existing clinical and pathological parameters; identifying the underlying biological parameters may be significant in improving their prognostic impact.

Advancement in the technologies and high throughput global profiling have led to the identification of a repertoire of candidate markers that can qualify as molecular prognosticators [17–20]. These markers can be predictive (to evaluate the likelihood of benefit from a specific clinical intervention) or prognostic (to evaluate the patient’s overall outcome). A cataloguing of the candidate markers that correlated with the aetiology and pathological parameters in a site-dependent manner, might provide insights into the molecules associated with treatment response/survival in each site and will therefore be more specific. This study proposed that molecular markers can serve as accurate adjuncts to existing prognostic parameters in head and neck cancers. Towards this effect, this study planned to mine the existing high throughput data to identify a panel of potential markers associated with etiological/clinical/pathological features in the three major sites in HNSCC; oral cavity, laryngopharynx and oropharynx.

## 2. Materials and methods

### 2.1 Data mining and meta-analysis

**Fig 1:**
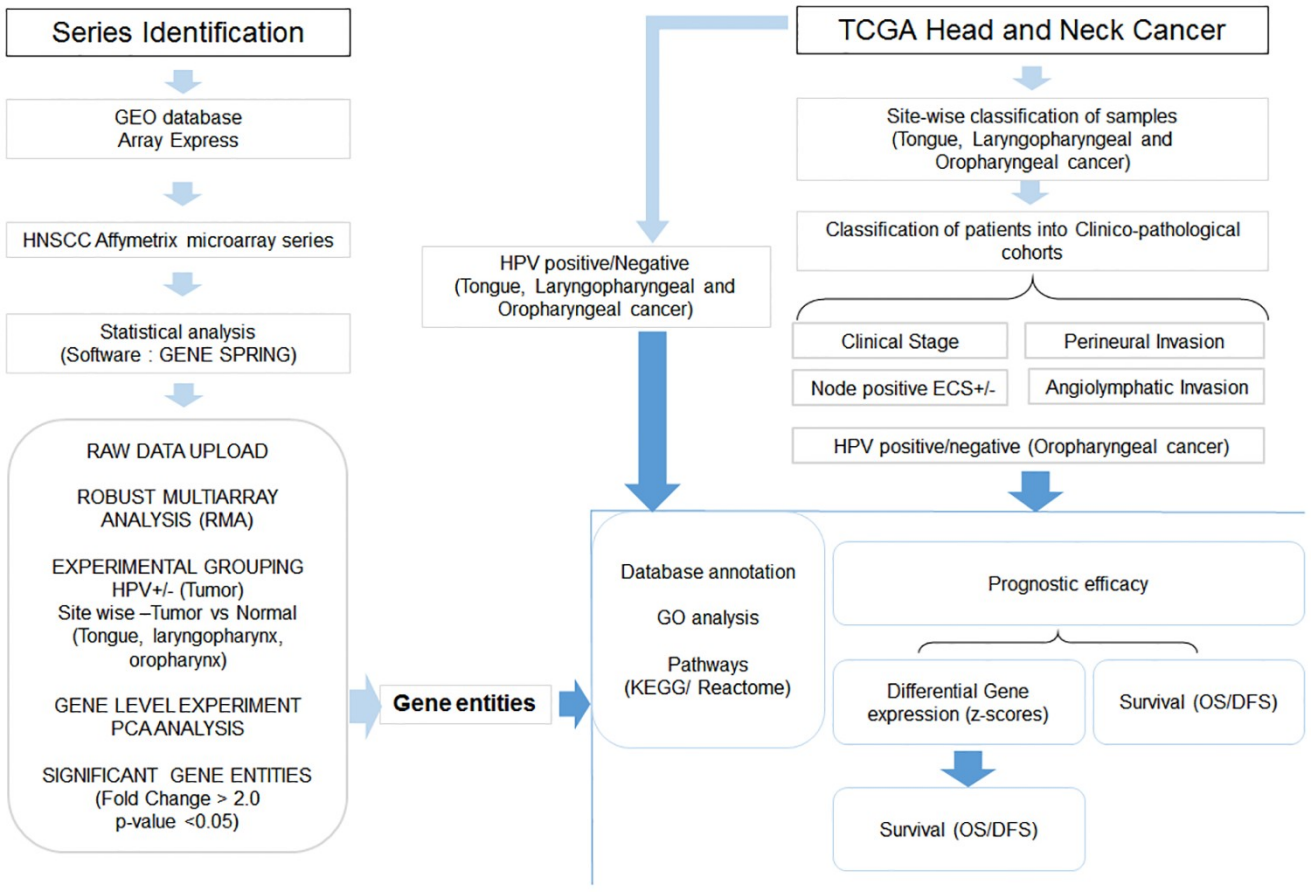
Over all work flow of site specific meta-analysis. Raw data (Affymetrix U133 Plus 2.0 microarray) of head and neck cancer series from publically available databases were downloaded and re-analyzed in Genespring statistical software. During analysis, post data upload onto the software, normalization (RMA) was carried out and samples grouped either into HPV positive/negative or based on sites (tongue, laryngopharyngeal and oropharyngeal cancer). In each site, further sample grouping was carried out into tumor and normal. Gene level experiment was performed in the software and PCA/clustering analysis was carried out to remove the discordant samples. Significant gene entities were identified (fold change >2.0, p-value <0.05) from each analysis/experiment. The annotation of the significant genes was carried out by TOPPFUN gene enrichment analysis (GO analysis/pathways). For validation, The Cancer Genome Atlas (TCGA) Head and Neck squamous cell carcinoma (HNSCC) cohort was classified based on HPV (HPV+/-) and site (tongue, laryngopharyngeal and oropharyngeal cancer). Patient cohorts in each site were further classified based on stage/pathological parameters and/or HPV. The significant gene entities identified from meta-analysis were validated in their respective cohorts; differential mRNA expression (z-score) and association with survival (OS/DFS) were the main endpoints.

The electronic search strategy included in the study is described as below (S1 Fig and Fig 1). Publicly available raw microarray data derived in head and neck cancer and normal sub sites of head and neck were mined from Gene Expression Omnibus (GEO) (NCBI; http://www.ncbi.nlm.nih.gov/geo/) and Array Express (EBI) [http://www.ebi.ac.uk/arrayexpress] [21]. A comprehensive search was carried out in the public databases for a period of January 2007 to December 2016. The datasets were searched using the keywords, “Head and Neck Cancer”, tongue squamous cell carcinoma, “oral tongue cancer”, “larynx”, laryngeal, “laryngeal squamous cell carcinoma”, “pharynx”, “pharyngeal”, “pharyngeal squamous carcinoma”, “oral cancer”“oropharyngeal” and oropharynx, while the search were filtered based on organism (“Homo sapiens” or “human”) and the assay systems (“RNA assay”, “array assay” or “expression profiling by array”). As a next step the identified series or datasets were screened for microarray performed by using Affymetrix Human Genome U133 plus 2.0 [Affymetrix Inc., California, USA], which is the most advanced microarray platform, with maximum number of probes. Eligible studies were finally selected based on the following inclusion and exclusion criteria for the identification of series. The inclusion criteria were i) experimental studies carried out by Affymetrix U133 plus 2.0 with available raw data ii) treatment naïve HNSCC patient’s iii) normal site of tongue, larynx pharynx and oropharynx. The exclusion criteria were i) studies carried out on HNSCC cell lines ii) studies with xenograft samples and iii) patient’s with treatment or recurrence or non-tissue sample (blood). The series selected were carried forward for meta-analysis.

Meta-analysis was carried out based on the stepwise protocol described previously [21] and as per the Preferred Reporting Items for Systematic Reviews and Meta-analysis (PRISMA) guidelines. Briefly, raw datasets (.CEL files) were downloaded from the database and meta-analysis carried out using the GeneSpring software (http://genespring-support.com) [v13.1, Agilent, California, USA] to avoid analytical bias from individual studies. The samples were grouped based on two parameters initially i) HPV association ii) site. For HPV based analysis, the tumor samples pertaining HPV status (positive/negative) were classified and re-analyzed as single experiment. In order to analyze individual sites (tongue, laryngopharyngeal and oropharynx), the samples were grouped into ‘tumor’ and ‘normal’ for re-analysis independently. For both the analytical pipelines, the samples were baseline transformed and normalized by Robust Multi-array Analysis (RMA). Gene level experiment was carried out by combining the arithmetic mean of all probes mapping to the same probe ID. Principal Component Analysis (PCA) was carried out for removal of outliers as a quality control check in the software (stratification of tumor and normal samples separately). Unpaired t-test (unequal variance) was carried out on the samples to obtain the significant gene entities on which fold change analysis was executed. Asymptotic p-value computation and Benjamini Hochberg FDR multiple testing correction was carried out to obtain gene entities with p-value <0.05 and fold change (FC) of > 2.0. The significant gene entities were extracted and exported to excel files for further downstream analysis.

### 2.2 Patient based validation in The Cancer Genome Atlas (TCGA)

Independent validation was carried out using TCGA database (http://www.cbioportal.org) [22,23]. The HNSCC patients (Provisional; n = 528 samples) in TCGA was categorized into different sites and further into sub cohorts based on the clinical information provided (HPV status, site, stage, and pathological parameters). The validation was carried out using two pipelines i) HPV associated meta-analysis gene list was validated in the HPV+ and HPV- cohort of TCGA ii) meta-analysis derived significant gene list obtained from each site was validated in the site-specific cohort in the TCGA HNSCC database. In both cases, the validation was carried out with regard to incidence of gene alteration (percentage alteration), differential profiling (mRNA expression status using z-score) and prognostic efficacy (correlation with survival) (Fig 1).

The mRNA expression z-scores (RNA Seq V2 RSEM) of significant gene entities were downloaded from TCGA HNSCC. The patient samples were categorized into HPV+/HPV- and within the sites further classified based on clinical and pathological condition. As a first tier analysis, the significance of the z-score distribution between the different groups; HPV, clinical stages (stage I-II vs stage III-IV) and pathological conditions (ALI, PNI, Node, ECS) was evaluated using the t-test unpaired unequal variance (p<0.05). The second tier analysis was carried out with the significant gene set to assess the heterogeneity of expression within each sub cohort of patient. This was assessed by evaluating the percentage of patients showing alteration in the gene (z-score threshold: ±2) in each cohort and difference in level of expression in the altered set (t-test unpaired unequal variance) (Fig 1).

Prognostic efficacy was assessed with markers identified at two levels i) mRNA z-score based differentials ii) markers with higher incidence of alterations in patients (>20%) (Fig 1). The markers were assessed for their survival impact in patients within the TCGA cohort categorized based on HPV and clinical stage (early and late stage), nodal metastasis (with and without extracapsular spread) and pathological (perineural invasion, angiolymphatic invasion, positive lymph node hematoxylin and eosin staining microscopy count, extracapsular spread pathologic) parameters within each site. For each analysis, TCGA samples without definite information for these parameters were excluded from the study. The contribution of these markers to the prognostic efficacy of these parameters (disease-free (DFS), overall survival (OS)) was assessed. The significant marker panel (p-value <0.04) were also evaluated for the prognostic efficacy in the different sub cohorts of patients in each site (Kaplan Meier plots; log rank test, p<0.05) (Fig 1).

### 2.3 Functional annotation of significant gene entities

Functional annotation of the significant gene entities was carried out by Gene Ontology [(GO; Biological Process (BP), Cellular Component (CC), and Molecular Function (MF)] and pathway analysis [Kyoto Encyclopedia of Genes and Genomes (KEGG), Reactome pathway] (p value: <0.05) using TOPPFUN (Transcriptome, ontology, phenotype, proteome, and pharmacome annotations based gene list functional enrichment analysis; https://toppgene.cchmc.org/enrichment.jsp) (Fig 1).

## 3. Results

### 3.1 Data mining and meta-analysis

A total of 8 datasets, wherein analysis was carried out using the Affymetrix platform (U133 plus 2.0) were included in this study (S1 File). The samples were categorized based on HPV status and then site. Among the series downloaded, two series had information on HPV status; a total of 97 tumor samples (HPV positive n = 12; Negative n = 85) were selected, 83 included in the final analysis after PCA/clustering analysis and set of 3258 gene entities were identified.

The tongue, laryngopharyngeal and oropharyngeal cancer samples (both tumor and adjacent normal) were separated out from the 8 series for analysis; a total of 192 samples (tumors (T): 154, normal (N): 38) were collected from the series, which included tongue (T = 62; N = 26), laryngopharyngeal (T = 46, N = 7) and oropharyngeal (T = 46; N = 5) cancers. The site-specific samples were grouped and analyzed separately in the GeneSpring for the identification of significant gene entities. PCA analysis removed the outliers and the samples that qualified were taken for further analysis in each site (tongue = 73, laryngopharyngeal = 48, oropharyngeal cancer = 43). Site-specific meta-analysis lead to the identification of the statistically significant gene entities (fold change (FC)> 2.0, p-value<0.05) specific to each site; 3508 in tongue samples, 4893 from laryngopharyngeal and 2386 from oropharyngeal cancers.

A comparison across the different sites indicated that 698 genes were common across all the 3 sites (S2 File). The significant gene entities from each site, when cross compared with the previously published database of 181 gene entities (up regulated: 168; down regulated: 13) [21] indicated that 66.3% (n = 119/181) of the genes in the database were identified in tongue cancer, while 109/181 genes (60.22%) were common with laryngopharyngeal subset and 95/181 genes (52.48%) were common with oropharyngeal cancer.

### 3.2 Molecular profile of HPV associated HNSCC

The 3258 gene entities (up = 2140; down = 1118) (S3A File) identified from analysis of HPV associated samples were annotated by GO analysis; the genes belonged to the extracellular matrix (GO: 0005201; 38.5%; n = 30) and structural molecule activity (GO: 0005198, 18.6%; n = 142) categories in molecular functions (MF). The major cellular component (CC) was complex of collagen trimers/collagen trimers (GO: 0098644/GO: 0005581; 30–47.8%), while among the biological processes (BP), positive regulation of reactive oxygen species (GO: 1903428, 32.2%; n = 19) and collagen catabolic process (GO: 0030574, 29.85%; n = 20) showed maximum representation (S3B File). Pathway analysis for the same gene entities (n = 3258) in TOPPFUN identified collagen assembly (38%; n = 23), PI3K-Akt signaling pathway (19.29%; n = 66), Focal adhesion (21.1%; n = 42) (KEGG) and GPCR ligand binding (19.56%; n = 89), Extracellular matrix organization (24.83%; n = 74) and Class A/1 (Rhodopsin-like receptors) (19.87%; n = 64) (Reactome) (S3C File) being the top pathways.

**Fig 2:**
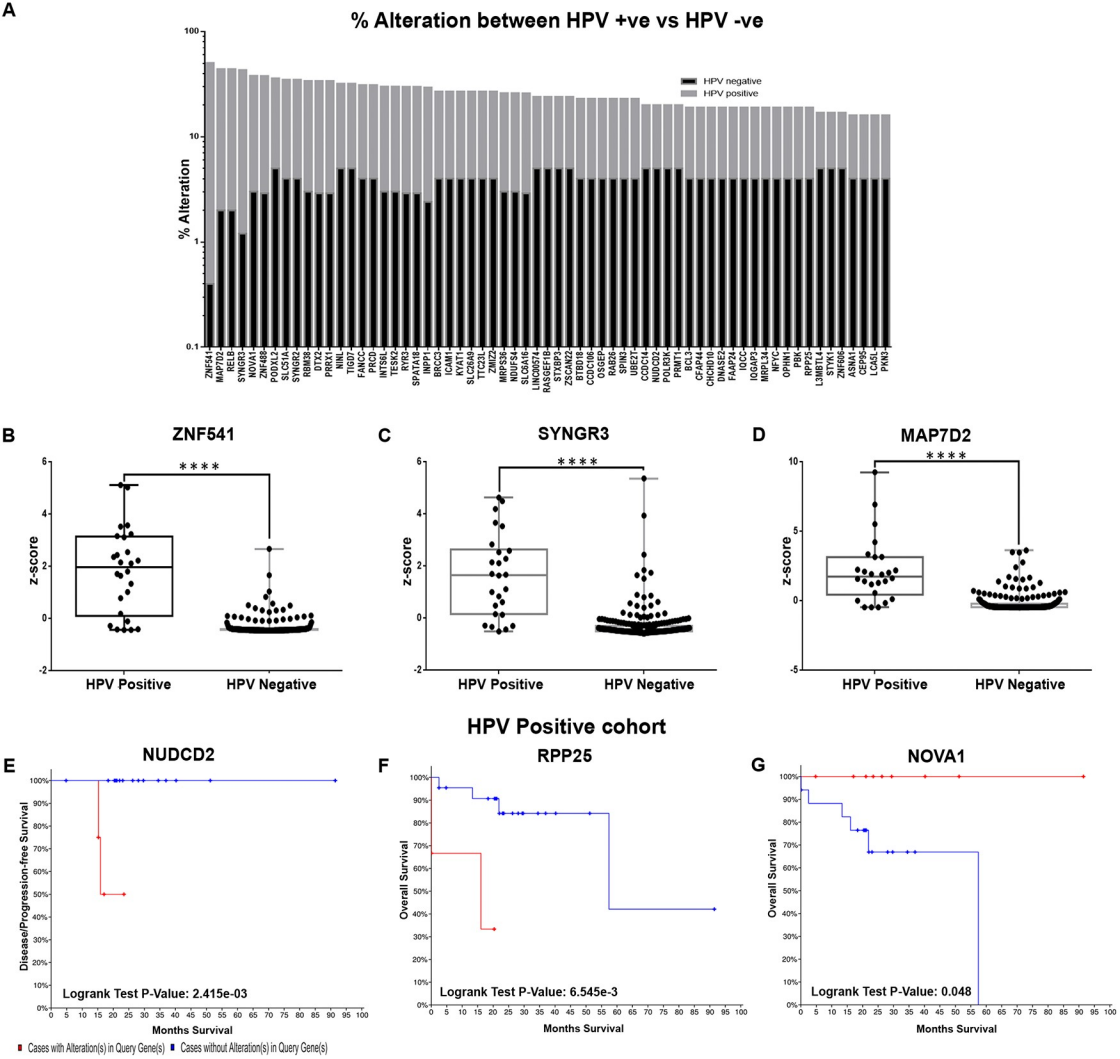
Marker profile in HPV+ HNSCC in terms of alterations, differential expression and prognostic efficacy. Comparison of the significant gene entities across HPV positive and negative cohort for their percentage alteration identified a total of 63 genes as highly altered in HPV positive cohort as compared to HPV negative cohort **(A)**. Assessment of expression levels (mRNA z-score) indicated that all these genes were significant differentials (p<0.05) between the two cohorts; the profile of top 3 genes (both alterations and z-score); ZNF541 (50%), SYNGR3 (42%), MAP7D2 (42%) in HPV positive TCGA cohort is represented as boxplots. **(B-D)**. The selected markers (n = 63) were analysed for their significant association with disease free and overall survival in patients with HPV positive cancer in TCGA. KM analysis indicated that NUDCD2 (**E**) was associated with DFS (p = 2.415e-03), RPP25 (**F**; p = 6.545e-3) and NOVA1 (**G**; good prognosticator, p = 0.048) were associated with OS. (P-value; **** p<0.00005).

The genes were validated in the TCGA HNSCC cohort with HPV information (n = 271; HPV+: 26, HPV-: 245) for their percentage alteration and the mRNA expression status; a total of 833 genes were altered in more than 5% and 267 genes in >10% of the cohort (S4A, S4B and S4C File). In order to document differences in alteration status between the HPV+/HPV- cohorts, the alteration status and expression levels of this subset (n = 267) were assessed separately in each cohort. Analysis to identify the genes exclusively altered in the HPV+ cohort, indicated that alterations in 63/267 genes were specific to the HPV+ (altered in >10% of cohort) with minimal (altered in <5% of the cohort) or no alterations in the patients negative for HPV (Fig 2A; S4D File). Among these, ZNF541 (Zinc Finger Protein 541) showed the highest alteration in 50% of HPV+ cases as compared to 0.5% in HPV- cases. The other top genes altered in HPV positive cohort includes SYNGR3 (Synaptogyrin 3, 42% vs 1.2%), MAP7D2 (MAP7 Domain Containing 2, 42% vs 2%), RELB (RELB Proto-Oncogene, NF-KB Subunit, 42% vs 2%), and ZNF488 (Zinc Finger Protein 488, 35% vs 3%) (Fig 2A). Assessment of the mRNA expression levels (z-score) indicated that this cohort of 63 genes showed significant differential expression between the two cohorts (p<0.04) (Fig 2B–2D; S4D File). The survival impact of these genes in patients positive for HPV (n = 26) was assessed; RPP25 showed association with overall survival (p = 6.545e-3), NUDCD2 (NudC Domain Containing 2) was associated with DFS (p = 2.415e-03), while NOVA1 (Neuro-Oncological Ventral Antigen 1) was a good prognosticator for overall survival in patients positive for HPV (p = 0.048) (Fig 2E–2G; S4E File).

### 3.3 Molecular prognosticators of tongue cancer

The significant genes identified from the meta-analysis of the tongue cancer cohort (n = 3508; up = 1753; down = 1755) (S5A File) were annotated based on the gene ontology classes. GO analysis (n = 3508) indicated that in molecular functions (MF), CXCR chemokine receptor binding (GO: 0045236; 64.7%; n = 11) and structural constituent of muscle (GO: 0008307; 63.4%; n = 26) category showed maximum representation. Among the cellular components (CC), TAP complex (GO: 0042825, 100%; n = 4), epidermal lamellar body (GO: 0097209, 100%; n = 4) and central spindling complex (GO: 0097149, 100%; n = 3) were highly represented, while in the biological process (BP), regulation of endodermal cell differentiation (GO: 1903224, 100%; n = 7), regulation of endodermal cell fate specification (GO: 0042663, 100%; n = 5) and regulation of protein kinase C activity (GO: 1900019, 100%; n = 4) showed maximum representation (S5B File). Pathway analysis carried out (n = 331) in TOPPFUN identified N-glycosylation by oligosaccharyl transferase (71.42%; n = 5) and proteasome (48.8%; n = 22) as the top pathways in KEGG with most number of overlapping genes. While in Reactome, the top pathways were Phosphorylation of Emi1 (83.3%; n = 5), Unwinding of DNA (66.67%; n = 8) and Platelet Adhesion to exposed collagen (53.8%; n = 7) (S5C File). The subset of significant genes (n = 3508) were assessed for their association with clinical/pathological parameters and survival.

#### 3.3.1 Markers associated with early stage tongue cancer

**Fig 3:**
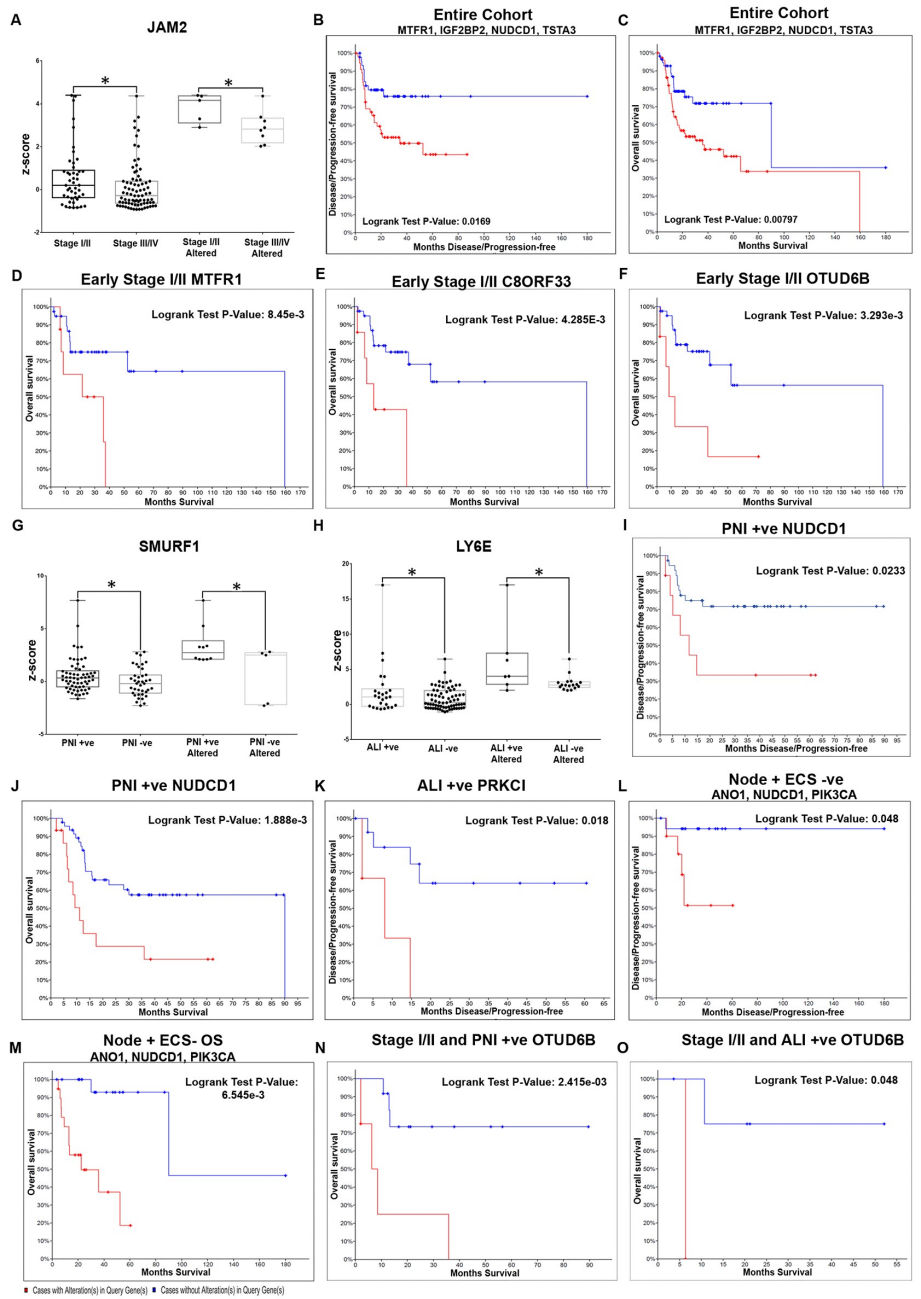
Molecular markers with significant differential expression and prognostic efficacy in tongue cancer. Significant differentials from tongue cancer meta-analysis were validated in terms of mRNA expression levels (z-score) across stage/pathological parameters in the TCGA tongue cancer cohort. In early stage tongue cancers, JAM2 showed a significantly upregulation (±2 fold) as compared to late stage **(A)**, with no effect on survival. Assessment of prognostic efficacy (genes >20% alterations) indicated that in the entire cohort (**B and C**), a combination of MTFR1, IGF2BP2, NUDCD1 and TSTA3 clearly showed a poor DFS (p = 0.0169) and OS (p = 0.00794). In early stage cancer **(D-F)**, MTRF1 (p = 8.450e-3), C8ORF33 (p = 4.285e-3) and OTUD6B (p = 3.293e-3) were individually associated with overall survival in early stage tongue cancer. Comparison of the differentials based on mRNA expression levels (z-score) between patients positive for pathological parameters (PNI, ALI, nodal metastasis) in tongue cancer TCGA cohort indicated that in the PNI+ cases, SMURF1 was upregulated **(G)**, while the gene LY6E was altered maximum in ALI+ cases (**H**). KM plot analysis in PNI+ patients **(I and J)** indicated that NUDCD1 showed an association with poor DFS (p = 0.023) and OS (p = 1.888e-3), while in ALI+ cases **(K)** PRKCI was associated with poor OS (p = 0.018). Analysis in the node +/ECS- cohort **(L and M)** of TCGA revealed that a combination of ANO1, NUDCD1 and PIK3CA was a poor prognosticator (DFS p = 0.0236 and OS p = 1.419e-4). Analysis in a further defined cohort of early stage tongue cancers positive for PNI or ALI, identified OTUD6B as a poor prognosticator (**N and O**). (P-value; * p<0.05).

In order to validate the gene entities in an independent cohort of patients, the TCGA patient cohort was used. The alteration status was evaluated in tongue cancer-specific patient cohort of TCGA (n = 132) to identify the highly prevalent genes; 331 genes were altered in at least 10% of the cohort, while 56 genes were altered in more than 20% (S5D File). To delineate the expression of these markers in different clinical stages, the TCGA tongue cancer patients were further classified into early/late stages and the expression profiling compared. mRNA z-score based differential profiling of the genes (n = 331) between the early and late stage cohorts indicated 14 genes as statistically significant differentials (p = >0.05) (S6A File); JAM2 (Junctional Adhesion Molecule) showing a significant alteration (FC>2 fold; p = 0.05) in the early stage patients (Table 1, Fig 3A). Evaluation of prognostic efficacy in early stage patients indicated that none of them were associated with survival.

**Table 1:** Differential mRNA z-score expression profiling between the altered cases in association with clinico-pathological parameters in tongue cancer.

			Percentage altered in cohort		
S.No	Parameter	Gene	Early stage	Late stage	p-value
1	Clinical stage	JAM2	10.63829787	9.756097561	0.049438
			**Positive**	**Negative**	
2	PNI	SMURF1	16.12903226	12.82051282	0.040467
3	ALI	LY6E	26.92307692	23.94366197	0.019605
		SUPT16H	15.38461538	7.042253521	0.009972
		MFN1	11.53846154	22.53521127	0.023416

**Fig 4:**
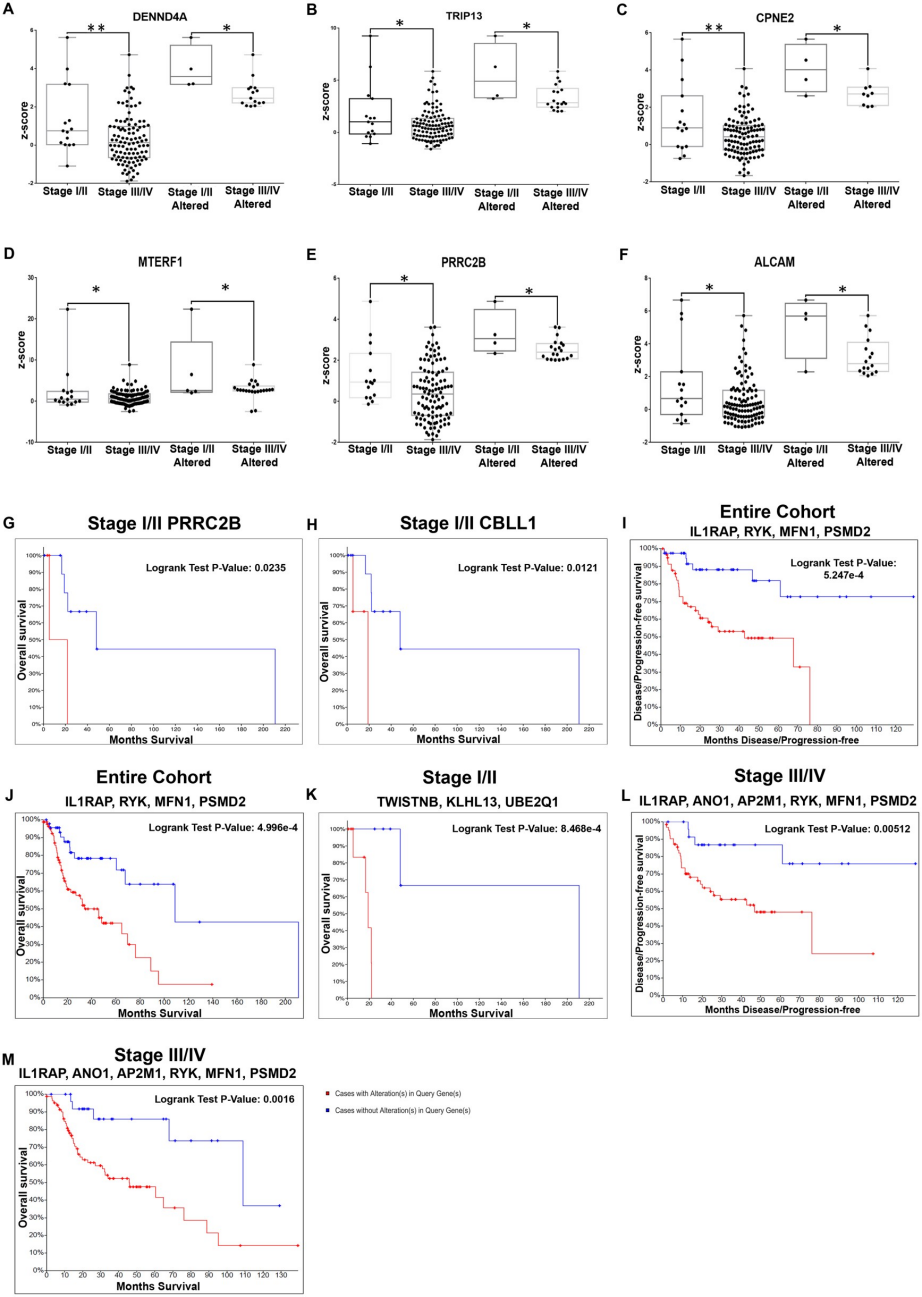
Molecular markers with significant differential expression and prognostic efficacy in laryngopharyngeal cancers. Meta-analysis based differentials in laryngopharyngeal cancers were validated in the TCGA cohort. mRNA expression levels (z-score) of the genes when compared stage-wise in the TCGA laryngopharyngeal cancer cohort identified 19 differentials in early stage cancers, the top six markers (DENND4A, TRIP13, CPNE2, MTERF1, PRRC2B and ALCAM) are represented here **(A-F)**. KM plot analysis of these genes in patients with early stage laryngopharyngeal cancers indicated PRRC2B (**G**; p = 0.023) and CBLL1 (**H**; p = 0.0121) showed an association with overall survival. Evaluation of the prognostic efficacy of the gene subset (altered in >20% of the patients) in the entire cohort of laryngopharyngeal cancers in TCGA identified the combination of IL1RAP, RYK, MFN1 and PSMD2 with low median survival in cases with alteration (DFS p = 0.0004; OS p = 0.0005) (**I and J**). Similar analysis in early stage patients **(K)** showed TWISTNB, KLHL13 and UBE2Q1 to be associated with overall survival (p = 8.468e-4), while in late stage patients, the combination of IL1RAP, ANO1, RYK, AP2M1, MFN1 and PSMD2 were associated with poor prognosis **(L and M)**. (P-value; * p<0.05; ** p<0.005).

As an additional effort to identify significant prognosticators, prognostic impact (overall/disease-free survival) was also investigated using the gene set altered in higher percentage of patients (>20%) in i) the overall tongue cancer cohort ii) early stage cohort iii) late stage cohort. In the overall tongue cohort, 4 genes showed an association for DFS and/or OS (p<0.05) individually and in combination. A combination of NUDCD1 (NudC domain containing 1) (Table 2), TSTA3 (Tissue specific transplantation antigen P35B), MTFR1 (Mitochondrial fission regulator 1) and IGF2BP2 (Insulin-like growth factor 2 mRNA binding protein 2) (S6B File) clearly demarcated the patients with poor survival in tongue cancer; patients with over-expression showed low median survival (35.91 vs 90.05 months; p = 0.007) and low DFS (34.76 vs NA months; p = 0.01) when compared with the cohort without alterations (Figs 3B and 4C).

**Table 2:** Prognosticators of survival (DFS and OS) in patients with tongue cancer in various clinic-pathological parameters.

S.No	Clinico-pathological parameter	Genes	Overall Survival Logrank Test P-Value	Disease Free Survival Logrank Test P-Value^#^
1	Entire cohort (differential expression)	NUDCD1, TSTA3, MTFR1 and IGF2BP2	0.007	0.01
2	Entire cohort	NUDCD1	0.0331	0.00958
3	Stage I-II	C8ORF33	4.28e-03	
		OTUD6B	0.0032	
		MTFR1	0.0084	
4	Stage III-IV	NUDCD1		0.0253
		CLPTM1L		0.0285
		MAPRE1		0.034
5	PNI +	NUDCD1	0.00187	0.0228
		TFG*	0.019	0.0331
		BRIX1*	0.0475	0.0437
6	ALI +	PRKCI		0.01
		WDR70*	0.0338	
		BRIX1*	0.0474	
7	Node+ ECS-	ANO1	0.00843	0.0133
		NUDCD1	0.00246	0.015
		ANO1, NUDCD1 and PIK3CA (Combination)	6.545e-3	0.048
8	Node+ ECS+	GMPS		0.0254
		TSTA3	0.0373	
9	Stage I-II and PNI +	OTUD6B	0.00166	0.0287
10	Stage I-II and ALI +	OTUD6B	0.0455	

*Genes which are good prognosticators in their respective clinic-pathological parameter,

^#^genes were sorted based on DFS.

Subsequent analysis to identify the markers that could distinguish patients of poor prognosis in early stage tongue cancer cohort of TCGA (stage I-II; n = 47) identified a three gene panel that independently demarcated patients with poor overall survival; MTFR1 (identified in the overall tongue cancer cohort) (p = 8.450e-3), C8ORF33 (Chromosome 8 open reading frame 33, p = 4.285e-3) and OTUD6B (OTU domain containing 6B, p = 3.293e-3) (Table 2; Fig 3D–3F; S6B File), although the marker combination was not significant. Interestingly, this analysis also revealed that NUDCD1 that was significantly correlated with all tongue cancer patients, was relevant (p<0.05) in advanced cancers (stage III-IV; n = 81) (S6B File), not in patients with early cancers.

#### 3.3.2 Markers with prognostic efficacy in patients with pathologically distinct tongue cancer

To identify the genes associated with severe pathological parameters in tongue cancer, the cohort of 331 genes was validated in the TCGA tongue cancer patients with perineural invasion (PNI, n = 65) or angiolymphatic invasion (ALI, n = 26). A panel of 17 genes were differentials in PNI+ or ALI+ as compared to the negative patients (p<0.05) (S6A File); SMURF1 (SMAD Specific E3 Ubiquitin Protein Ligase 1) (Fig 3G) was significantly altered in PNI+ patients and a panel of 3 genes were significantly altered in ALI+ patients (FC>±2; p<0.05) (Table 1; Fig 3H). None of these genes showed a survival impact. Prognostic efficacy assessed with genes having alterations in a larger cohort of patients (>20%) in the multiple pathological sub-cohorts (PNI+, ALI+, node+, node+/ECS+ and node+/ECS-) identified 4 genes in PNI+ patients (n = 62); NUDCD1 (Fig 3I and 3J), TFG (TRK-Fused Gen), TMEM267 (Transmembrane Protein 267) and BRIX1 (BRX1, Biogenesis of Ribosomes) as significant in survival prediction (OS and/or DFS; p<0.05), while PRKCI (Protein kinase C, iota) (Fig 3K) WDR70 (WD repeat domain 70) and BRIX1 were predictors in patients with ALI+ (n = 26) (Table 2; S6B File) although the marker combination was not significant in both the parameters.

A similar investigation into markers associated extracapsular spread (ECS) in tongue cancer indicated a panel of 17 differentials in node+ ECS+ patients (p<0.05) (S6A File). However, none of these genes were significantly altered or showed any significant survival impact. In next level analysis of the prognostic efficacy of genes with alterations in >20% of patients of node positive patients (n = 68), NUDCD1, PUF60 (poly-U binding splicing factor 60KDa), ANO1 (Anoctamin 1) and RHOA (Ras Homolog Family Member A) showed an association with OS/DFS (S6B File). These genes showed no correlation with survival in negative patient cohort. Analysis of the sub cohorts with/without ECS (ECS+: 24; ECS-: 39) indicated that NUDCD1 and ANO1 were significant prognosticators (Table 2; S6B File) in the ECS- cohort. In this sub-cohort, patient with overexpression of the marker combination, ANO1, NUDCD1 and PIK3CA (Phosphatidylinositol-4, 5-Bisphosphate 3-Kinase Catalytic Subunit Alpha) showed low median survival (22.34 vs 90.05 months; p = 0.00014) (Fig 3L and 3M) indicating their relevance in cohort which is otherwise considered to have a good prognosis. Further in the ECS+ patients (n = 24), wherein presence of ECS itself indicated poor prognosis, GMPS (Guanine monophosphate synthetase) (DFS; p = 0.025), TSTA3 (OS; p = 0.0373) were additional prognosticators (Table 2; S6B File).

Multiple level classification including stage and pathological parameters indicated that in the early stage patients with poor prognosis (PNI+ and/or ALI+), OTUD6B was an additional prognosticator (p<0.05) (Table 2; Fig 3N and 3O).

### 3.4 Molecular prognosticators of laryngopharyngeal cancers

A total of 4893 genes (up = 2017; down = 2876) from laryngopharyngeal samples with FC >2 and a p-value of <0.05 were identified (S7A File). GO analysis of the significant (n = 4893) genes indicated that among molecular functions (MF), DNA replication origin binding (GO: 0003688) showed maximum representation (88.88%; n = 8) followed by CXCR chemokine receptor binding (GO: 0045236) (64.70%; n = 11). Condensed nuclear chromosome outer kinetochore (GO: 0000942, 100%; n = 4) was the major cellular component, while in the biological process (BP), regulation of endodermal cell differentiation (GO: 1903224, 100%; n = 7) and regulation of endodermal cell fate specification (GO: 0042663, 100%; n = 5) showed maximum representation (S7B File). Pathway analysis identified DNA replication (52.77%; n = 19), Cell cycle (45.16%; n = 56), condensation of Prometaphase Chromosomes (88.18%; n = 9), Zinc influx into cells by the SLC39 gene family (80%; n = 8) and Unwinding of DNA (75%; n = 9) (S7C File) as the top relevant pathways.

#### 3.4.1 Markers associated with early stage laryngopharyngeal cancers

These significant gene entities were validated in the TCGA HNSC laryngopharyngeal specific samples (n = 127) in terms of prevalence, differential expression and prognostic efficacy. Assessment of alteration status identified that 701 genes were altered in mRNA expression in >10% of patients and 137 genes were altered in at least 20%. The expression of the 701 genes was looked into in the TCGA laryngopharyngeal subset (n = 127) classified based on stage; 82 genes were differentially regulated (p<0.05) between the early/late stage laryngopharyngeal cancers, out of which 19 genes showed a significant alteration (FC >±2) in the expression levels (Table 3; Fig 4A–4F; S8A File). These differential genes were assessed for their impact on overall and disease free survival; PRRC2B (Proline Rich Coiled-Coil 2B, p = 0.0235) and CBLL1 (Cbl proto-oncogene, E3 ubiquitin protein ligase-like 1, p = 0.0121) impacted overall survival of early stage laryngopharyngeal patients (Fig 4G and 4H; S8B File).

**Table 3:** Differential mRNA z-score expression profiling between the altered cases in association with clinico-pathological parameters in laryngopharyngeal cancer.

			Percentage altered in cohort		
S.No	Parameter	Gene	Early stage	Late stage	p-value
1	Clinical stage	MTERF1	33.33333333	20.56074766	0.039969451
		DENND4A	26.66666667	14.01869159	0.011957913
		TRIP13	26.66666667	16.82242991	0.016278625
		ALCAM	26.66666667	14.95327103	0.022132352
		CPNE2	26.66666667	8.411214953	0.027141198
		PRRC2B	26.66666667	18.69158879	0.027273281
		P3H2	20	17.75700935	0.000163948
		ORC5	20	22.42990654	0.003092435
		CBLL1	20	20.56074766	0.005484005
		ANKIB1	20	17.75700935	0.011002909
		SMIM13	20	12.14953271	0.011095341
		NUP155	20	16.82242991	0.037923343
		RBM48	13.33333333	17.75700935	6.57699e-05
		SKP2	13.33333333	14.01869159	0.00127573
		STK38L	13.33333333	10.28037383	0.001602153
		PON2	13.33333333	13.08411215	0.00565697
		CYP3A5	13.33333333	12.14953271	0.007557119
		CDK6	13.33333333	9.345794393	0.015929102
		WAPL	13.33333333	8.411214953	0.020646728
		**Gene**	**Positive**	**Negative**	p-value
2	PNI	RSRC1	51.72413793	46.15384615	0.006797288
		PIK3CA	48.27586207	38.46153846	0.034488768
		DCUN1D1	44.82758621	28.84615385	0.047521591
		FYTTD1	37.93103448	21.15384615	0.03346135
		SLC4A1AP	20.68965517	5.769230769	0.017492707
		CWC22	17.24137931	19.23076923	0.015213151
		COMMD2	10.34482759	5.769230769	0.011896098
3	ALI	PAK2	45.94594595	35.55555556	0.011671511
		FXR1	43.24324324	20	0.043115543
		MFN1	37.83783784	31.11111111	0.005977193
		SLC4A1AP	16.21621622	6.666666667	0.012880852
4	Node positive ECS	EPS8	19.23076923	38.0952381	0.032931489

Prognostic efficacy was assessed for genes (>20%; n = 137) in i) entire cohort (n = 127) of laryngopharyngeal patients ii) early stage patients. Assessment in entire cohort identified 27 significant predictors of survival (OS and/or DFS) (Table 4; S9A File); patients with overexpression of IL1RAP (Interleukin 1 Receptor Accessory Protein), RYK (Receptor-like tyrosine kinase), PSMD2 (Proteasome (prosome, macropain) 26S subunit, non-ATPase, 2) and MFN1 (Mitofusin 1) showed low median survival (34.07 vs 108.81 months; p = 0.0005) and low DFS (42.77 vs NA months; p = 0.0004) when compared with the cohort without alterations (Fig 4I and 4J; S9B File). Assessment of the prognostic efficacy in the early stage laryngopharyngeal samples (I-II; N = 15) revealed a total of 12 gene predictor panel completely unique to this cohort (Table 4; S9A File). Among these genes, the combination of TWISTNB (TWIST Neighbor), KLHL13 (Kelch-like 13) and UBE2Q1 (Ubiquitin-conjugating enzyme E2Q family member 1) and WBP11 (WW Domain Binding Protein 11) proved to be accurate survival predictors (low median survival (18.96 vs 210.81 months; p = 0.0008) (Fig 4K; S9B File). As observed in the case of tongue, genes associated with overall cohort, IL1RAP, ANO1, RYK, AP2M1, MFN1 and PSMD2 were significant prognosticators only in the late stage not in early cancers. (Table 4; Fig 4L and 4M; S9A and S9B File).

**Table 4:** List of significant genes with both disease free and overall survival of different clinic-pathological parameters in TCGA laryngopharyngeal cohort.

S.No	Clinico-pathological parameter	Genes	Overall Survival Logrank Test P-Value	Disease Free Survival Logrank Test P-Value^#^
**1**	Entire cohort	IL1RAP	0.015	3.47e-06
		RYK	0.00979	7.25e-04
		AP2M1	0.0288	0.00106
		ANO1	0.0465	0.00153
		MFN1	0.0094	0.0081
		PSMD2	8.94e-04	0.00994
		PIK3CA	0.0449	0.0145
		TRMT12	0.0422	0.0346
		IGF2BP2	0.0277	0.04
		ZNF12	0.0426	0.044
		IL1RAP, RYK, MFN1 and PSMD2 (Combination)	5.25e-04	5.00e-04
2	Stage I-II*	TWISTNB	0.00166	
		KLHL13	0.00173	
		UBE2Q1	0.0037	
		WBP11	0.0108	
		ATP1B1	0.0235	
		ZNF687	0.0253	
		FXR1	0.0453	
		PFN2	0.0453	
		EPS8	0.0453	
		WDR53	0.0453	
		SDE2	0.0465	
		TERF1	0.0453	
		TWISTNB, KLHL13, UBE2Q1 and WBP11(combination)	8.47e-04	
3	Stage III-IV	IL1RAP	0.00396	1.32e-07
		ANO1	0.0186	5.25e-04
		RYK	0.0106	0.00175
		AP2M1	0.00474	0.00356
		MFN1	0.00881	0.0148
		PSMD2	0.0012	0.0153
		TRMT12	0.0353	0.0275
		IGF2BP2	0.0207	0.0429
		IL1RAP, ANO1, RYK, AP2M1, MFN1 and PSMD2 (combination)	0.0016	0.00512
**4**	PNI +	AP2M1	0.0133	0.0259
**5**	ALI +	RYK	0.00245	0.00717
		IL1RAP	0.0333	0.0125
		AP2M1	0.0359	0.035
		RYK and IL1RAP (combination)	6.79e-04	0.0209
**6**	Node+ ECS+	STK3	0.00163	0.00106
		TMEM267	0.00298	0.00121
		CLPTM1L	0.0489	0.0118
		PSMD2	0.00504	0.0175
		STK3, TMEM267 and PSMD2 (combination)	6.11e-04	0.00401
**7**	Node+ ECS-	VKORC1L1	0.0323	0.00545

*In stage I-II all the genes were associated with only overall survival,

^#^genes were sorted based on DFS.

#### 3.4.2 Markers correlating with pathological parameters

**Fig 5:**
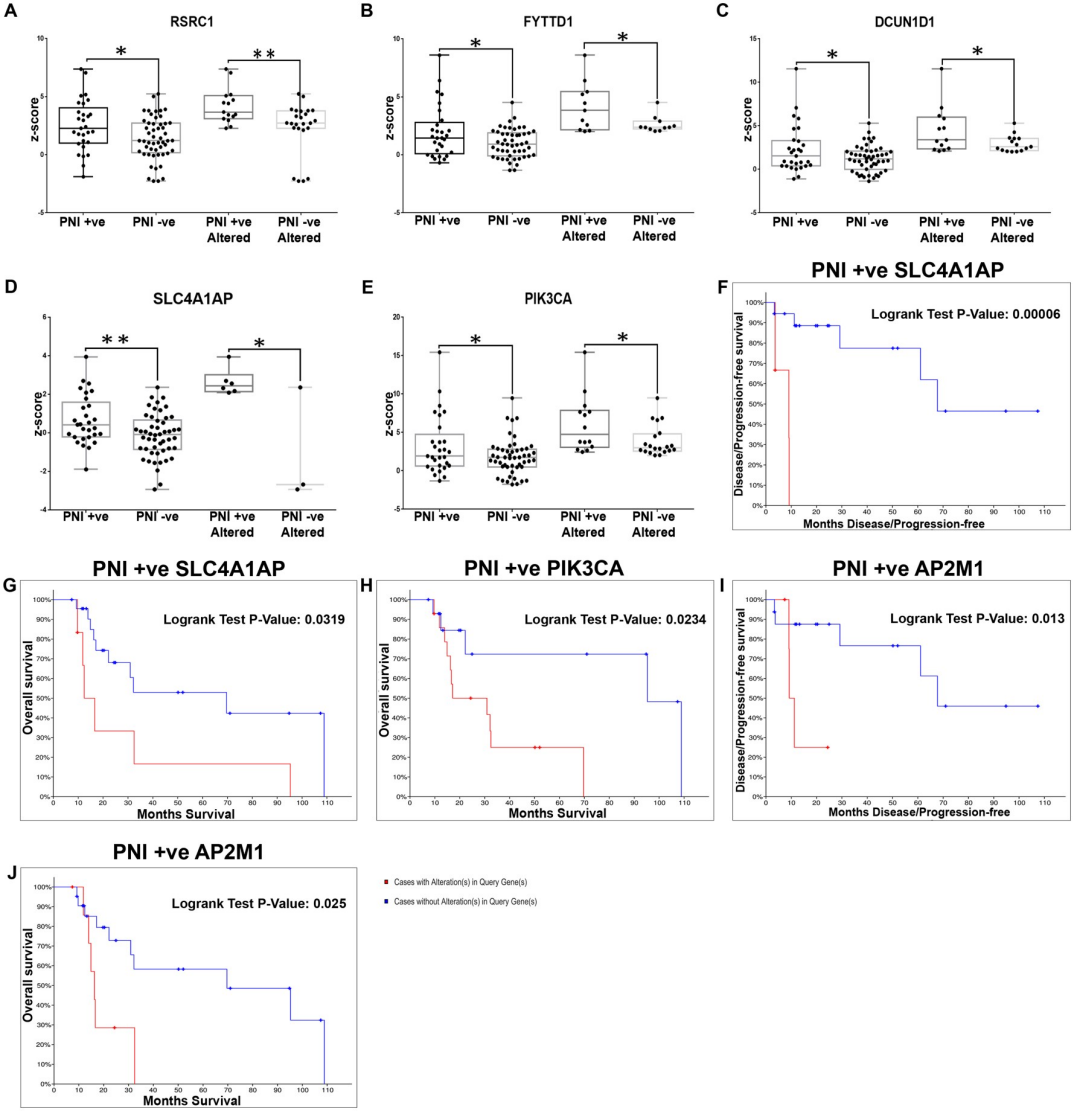
Molecular markers with significant differential expression and prognostic efficacy in laryngopharyngeal cancers with perineural invasion. Validation of the differentials identified in the laryngopharyngeal cancer within the TCGA cohort of patients with perineural invasion identified 7 gene panel based on differences in mRNA expression levels (z-score), the top 5 based on percentage alteration being RSRC1, FYTTD1, DCUN1D1, SLC4A1AP and PIK3CA was upregulated in altered cases of PNI positive patients **(A-E)**. From among this cohort, SLC4A1AP showed an association in both DFS (**F**; p = 0.00006569) and OS (**G**; p = 0.0319), whereas PIK3CA was associated with overall survival (**H**; p = 0.0234). Assessment of the prognostic efficacy of the genes with alterations >20% identified AP2M1 as the sole marker with survival impact on both OS (**I**; p = 0.013) and DFS (**J**; p = 0.025). (P-value; * p<0.05; ** p<0.005).

The genes (n = 701) were assessed for differential expression in the laryngopharyngeal patients triaged based on pathological categories. In the PNI+ subset (n = 29), 7 genes showed statistically significant alteration as compared to PNI- patients (p<0.05) (Fig 5A–5E; S8A File); SLC4A1AP (Solute Carrier Family 4 Member 1 Adaptor Protein, OS; p = 0.03 and DFS; p = 6.57e-05) and PIK3CA (OS; p = 0.0234) added to the survival impact of perineural invasion in PNI+ cases. (Fig 5F–5H; S8B File). Additional analysis to identify independent prognosticators in the PNI+ laryngopharyngeal cancer patients, identified AP2M1 (Adaptor Related Protein Complex 2 Subunit Mu 1) (Table 4; Fig 5I and 5J) as the most significant predictor (DFS and OS) in addition to a panel of 12 genes which could predict disease free/overall survival (S9A File).

**Fig 6:**
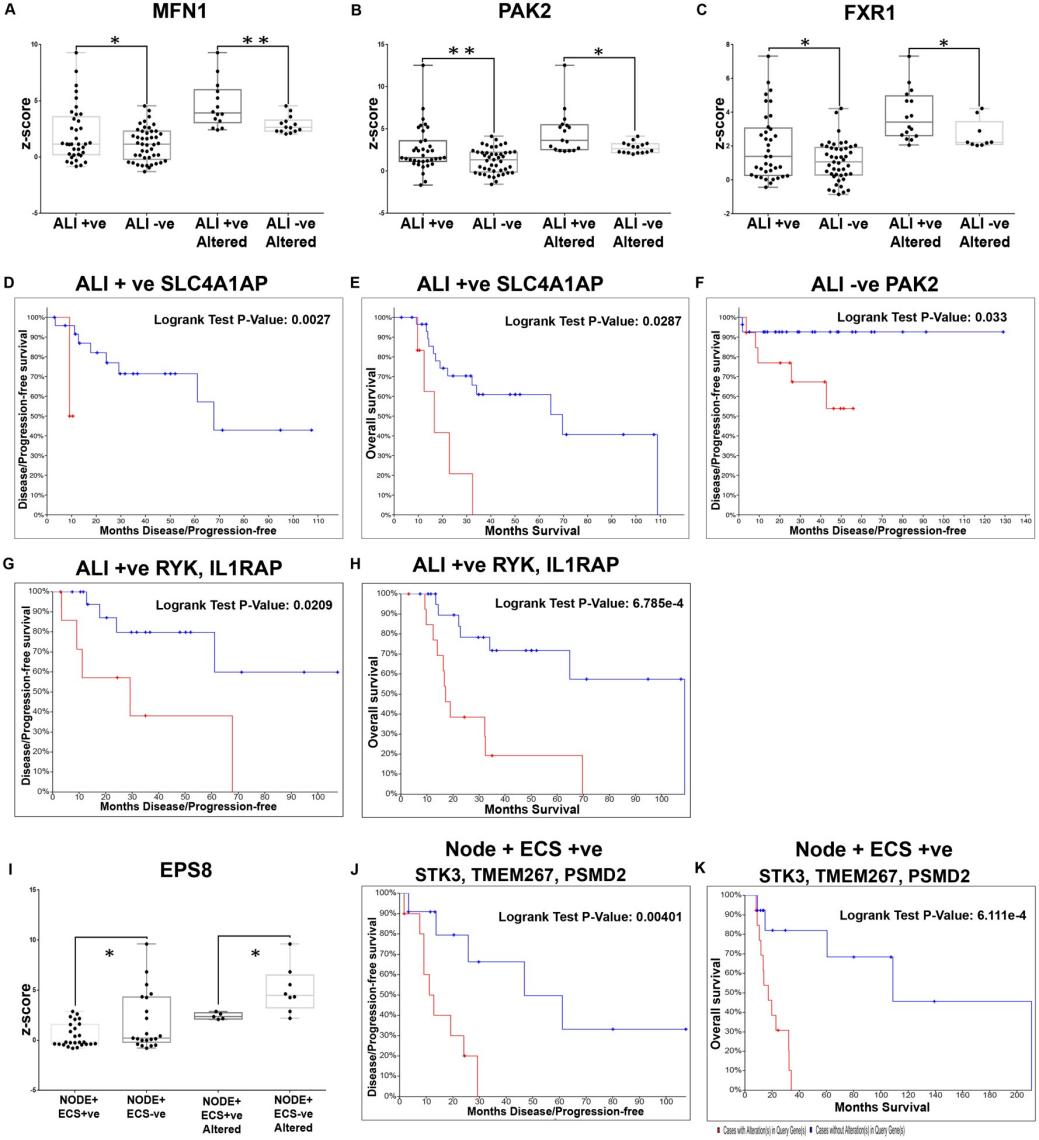
Molecular markers with significant differential expression and prognostic efficacy in laryngopharyngeal cancers with angiolymphatic invasion and nodal metastasis. Validation of the differentials identified in the laryngopharyngeal cancer within the TCGA cohort of patients with angiolymphatic invasion identified 4 genes with significant difference in mRNA expression levels (z-score) in TCGA cohort laryngopharyngeal cancer with ALI; the top three genes being MFN1, PAK2 and FXR1 (**A-C**). Among this cohort, SLC4A1AP showed an association in both OS (**D**; p = 0.002785) and DFS (**E**; p = 0.0287) of ALI+ patients, while PAK2 showed association with ALI- in DFS alone (**F**; p = 0.033). Prognostic assessment of the subset of meta-analysis based differentials altered in >20% of the patients identified the combination of RYK and IL1RAP to be associated with poor survival **(G and H)**. EPS8 was the only differential in node in node+ECS- patients with no survival impact **(I)** validation of the differentials associated with node positive with/without ECS indicated that EPS8 was the only differential (downregulated) in node+/ECS+ cohort with no association with survival. Prognostic efficacy (subset altered in >20% patients) indicated that STK3, TMEM267 and PSMD2 were the prognosticators of poor survival **(J and K)**. (P-value; * p<0.05; ** p<0.005).

In the ALI+ set (n = 37), 4 genes [MFN1, PAK2 (P21 (RAC1) Activated Kinase 2), SLC4A1AP and FXR1 (Fragile X mental retardation syndrome-related protein 1)] were significant differentials (Table 3; Fig 6A–6C; S8A File), with SLC4A1AP being a determinant of both OS (p = 0.0027) and DFS (p = 0.0287) (Fig 6D and 6E). PAK2 was associated with disease free survival (p = 0.033; Fig 6F) in ALI- cases (S8B File). Additional analysis (genes with alterations in >20% patients) in ALI+ patients revealed that RYK, AP2M1 and IL1RAP that relevant in the overall cohort were significantly predictors of OS and DFS (Table 4; S9A File). Among these markers, the overexpression of the combination of RYK and IL1RAP in ALI+ patients, led to low median survival (17.12 vs 108.87 months; p = 0.0006) and low DFS (29.2 vs NA months; p = 0.02) when compared with the cohort without alterations (Fig 6G and 6H; S9B File). The assessment of differential prognosticators of early stage laryngopharyngeal patients with PNI/ALI+ was not carried out due to less number of patients in this cohort.

EPS8 (Epidermal growth factor receptor kinase substrate 8) was the sole marker that showed significant upregulation (>2 fold) in the patients with node+ ECS+ as compared to node+ ECS- patients (Fig 6I), but without any impact on the survival of the patients. In node positive laryngopharyngeal patients (n = 54) prognostic efficacy was also assessed with genes having increased alterations (>20%), 17 markers were poor predictors of survival (S9A File); among which IL1RAP and PSMD2 were predictors in the entire cohort. Further, assessment in node+ ECS+ patients (n = 26), identified 21 genes associated with DFS and/or OS; PSMD2 being significant in this sub cohort also (S9A File). A combination panel of STK3 (Serine/Threonine Kinase 3), TMEM267 and PSMD2 clearly demarcated the patients [node+ ECS+] with poor median survival (17.12 vs 108.87 months; p = 0.0006) and low DFS (11.2 vs 46.81 months; p = 0.004) when compared with the cohort without alterations (Fig 6J and 6K, S9B File). In [node+ ECS-] patients (n = 21), who are known to have a better prognosis than the [node+ ECS+] patients, alterations in VKORC1L1 (Vitamin K epoxide reductase complex, subunit 1-like 1), KLHL12 (Kelch-like 12), PAIP1 (poly (A) binding protein interacting protein 1), CBLL1 and PUS7 (Pseudouridine Synthase 7) could predict OS/DFS (Table 4; S9A File).

### 3.5 Molecular prognosticators of oropharyngeal cancers

Meta-analysis identified 2386 genes (Up = 1767; Down = 619) in oropharynx samples (S10A File) (n = 51). GO analysis carried out to evaluate the functional classes indicated that in molecular functions (MF), extracellular matrix constituent conferring elasticity (GO: 0030023) category showed maximum representation (100%; n = 6) followed by 2'-5'-oligoadenylate synthetase activity (GO: 0001730, 100%; n = 4). Among the cellular components (CC), laminin-1 complex (GO: 0005606 100%; n = 3) and collagen type VI trimer (GO: 0005589, 100%; n = 3) classes were highly represented, while in the biological process (BP), adenine biosynthetic process (GO: 0046084, 100%; n = 4) and adenine metabolic process (GO: 0046083, 100%; n = 4) showed maximum representation (S10B File). Analysis also identified N-glycosylation by oligosaccharyl transferase (71.42%; n = 5), Guanine ribonucleotide biosynthesis IMP = > GDP,GTP (46.15%; n = 6), Glycolysis (Embden-Meyerhof pathway), glucose = > pyruvate (44%; n = 25) and ECM-receptor interaction (36.58%; n = 30) were significant pathways in KEGG, while TFAP2 (AP-2) family regulates transcription of cell cycle factors (100%; n = 5), Antagonism of Activin by Follistatin (100%; n = 4) and Phosphorylation of proteins involved in the G2/M transition by Cyclin A:Cdc2 complexes 100%; n = 3) were major pathways in Reactome (S10C File).

Validation in the TCGA oropharyngeal cancer cohort (n = 32) indicated that 404 genes were altered at the mRNA expression level in at least 10% of the cohort, while a sub-set of 73 genes showed a high prevalence and were altered in >20% of the patients (S11A File). Analysis of these genes in terms of z-score analysis and prognosis in correlation with stage/pathological parameters and survival analysis was not carried out due to the low number of samples in the sub-categories.

#### 3.5.1 Markers correlating to HPV-associated oropharyngeal cancers

**Fig 7:**
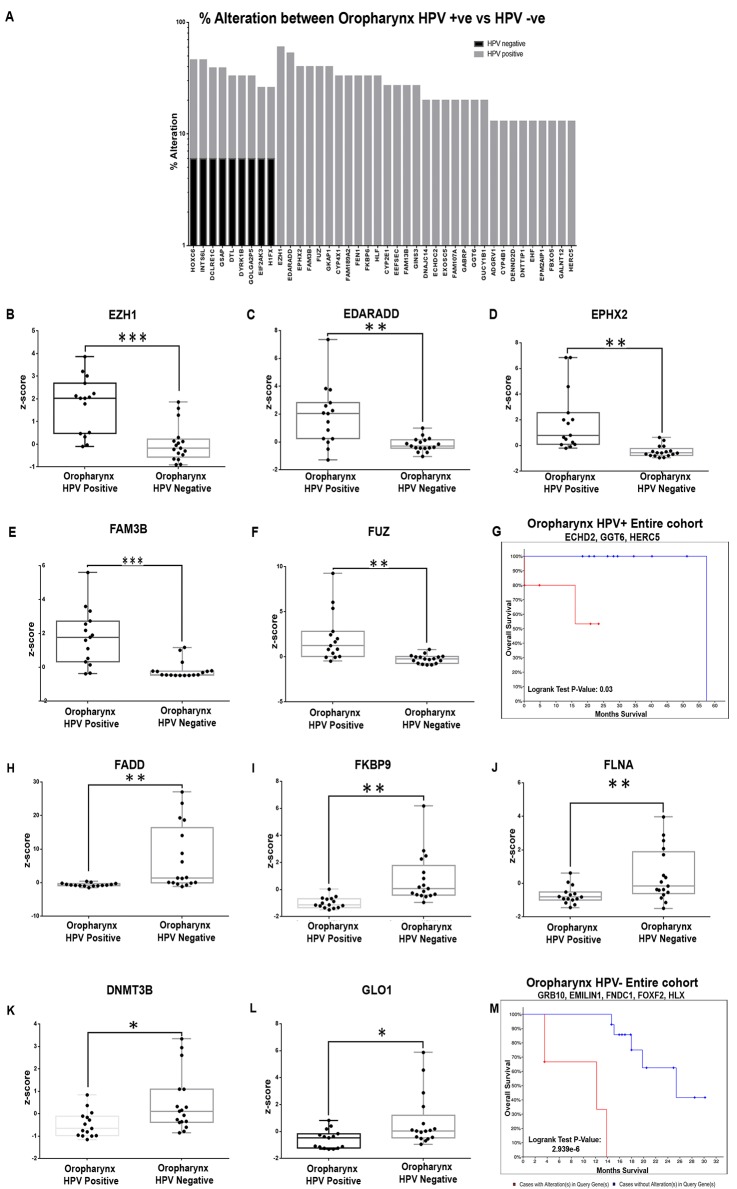
Marker profile of HPV+/HPV- oropharyngeal cancers in terms of percentage alterations, differential expression and survival. The significant gene entities identified from the meta-analysis of oropharyngeal cancers were compared with the oropharyngeal HPV positive and negative cohort from TCGA. A total of 31 genes showed to be highly altered in HPV positive cohort with minimal/ no alteration in negative cohort. The top 5 genes altered exclusively in HPV+ cohort included EZH1 (60%), EDARADD (42%), EPHX2 (42%), FAM3B (42%) and FUZ (35%) (**A**). Differential profiling of these genes based on mRNA expression levels (z-score) indicated that these genes showed a significantly upregulation in oropharyngeal HPV+ cases **(B-F)**. The selected markers (n = 31) were analysed for their significant association with disease free and overall survival in oropharyngeal HPV+ cohort in TCGA; KM plot analysis identified that a combination of ECHD2, GGT6 and HERC5 (all genes uniquely altered in the HPV+ cohort) showed a significant association with overall survival (**G**; p = 0.0321). Similar validation of the genes in the HPV- cohort identified 27 genes altered; mRNA expression levels (z-score) of the top 5 genes [FADD (41%), FKBP9 (24%), FLNA (24%) DNMT3B (18%) and GLO1 (18%)] (**H-L**) indicated that they were significantly upregulated in the HPV+ cohort. Assessment of prognostic efficacy in HPV- cohort showed that a combination of GRB10, EMILIN1, FNDC1, FOXF2 and HLX showed a significant association with overall survival (**M**; p = 2.939e-6). (P-value; * p<0.05; ** p<0.005; *** p<0.0005).

The markers were assessed for their relevance in HPV associated oropharyngeal cancers; the gene set was validated for alterations/survival impact in the TCGA oropharyngeal cohort segregated according to HPV status. Among the 404 gene set, 128 genes were altered in positive cohort (n = 15), while 131 were altered in the negative cohort (n = 17) (S11B and S11C File). Comparison of the alteration status across the two cohorts indicated that 51 genes in HPV+ and 55 in HPV- were either altered only in the respective cohorts or showed a high percentage alteration (S11D File). Assessment of the mRNA expression level (z-score) differences in these genes indicated that 31 genes in HPV+ cohort (Fig 7A) and 27 genes in HPV- cohort showed significant difference in the expression levels between the two cohorts (p<0.04).

The significantly altered genes in the HPV+ cohort include EZH1 (Enhancer Of Zeste 1 Polycomb Repressive Complex 2 Subunit, 60% vs 0%), EDARADD (Ectodysplasin-A Receptor-Associated Adapter Protein Associated Death Domain, 42% vs 1.2%), EPHX2 (Epoxide Hydrolase 2, 42% vs 2%), FAM3B (Family With Sequence Similarity 3 Member B, 42% vs 2%), and FUZ (Fuzzy Planar Cell Polarity Protein, 35% vs 3%) (Fig 7B–7F). Assessment of prognostic efficacy in HPV+ cohort indicated that although these genes were differentials, they did not have a survival impact. Among the genes altered in >20% of patients, ECHDC2 (Enoyl-CoA Hydratase Domain Containing 2, p = 2.22e-03), GGT6 (Gamma-Glutamyltransferase 6, p = 2.22e-03), HERC5 (HECT And RLD Domain Containing E3 Ubiquitin Protein Ligase 5, p = 0.018), EEFSEC (Eukaryotic Elongation Factor, Selenocysteine-TRNA Specific, p = 0.013), FEN1 (Flap Structure-Specific Endonuclease 1, p = 0.02), ADGRV1 (Adhesion G Protein-Coupled Receptor V1) and GINS3 (GINS Complex Subunit 3) were associated with overall survival/DFS (p = 0.03); a combination of the genes ECHD2, GGT6 and HERC5 showed a significant association with overall survival in entire cohort (p = 0.0321) (Fig 7G; S12A File).

A similar differential analysis in the HPV- cohort indicated a panel completely distinct from the HPV+ oropharyngeal cancers; FADD (Fas Associated via Death Domain, 41%), FKBP9 (FKBP Prolyl Isomerase 9, 24%), FLNA (Filamin A, 24%) and DNMT3B (DNA Methyl transferase 3 Beta, 18%) with high prevalence (Fig 7H–7L). Assessment of prognostic efficacy of the differentials (n = 55) indicated that in HPV- cohort, FARSA (Phenylalanyl-TRNA Synthetase Subunit Alpha) showed an association with both overall and disease free survival (p<0.03), while GRB10 (Growth Factor Receptor Bound Protein 10), EMILIN1 (Elastin Microfibril Interfacer 1), FNDC1 Fibronectin Type III Domain Containing 1), FOXF2 (Forkhead Box F2), HLX (H2.0 Like Homeobox), GTF2F1 (General Transcription Factor IIF Subunit 1) were associated either with overall or disease free survival (p< 0.01). A combination of the genes GRB10, EMILIN1, FNDC1, FOXF2 and HLX showed a significant association with overall survival (p = 2.939e-6) (Fig 7M; S12B File).

## Discussion

Heterogeneity in treatment outcome is a major aspect of concern in solid tumors. In head and neck cancers (HNSCC), diverse behavioral and molecular etiologies drive tumors in anatomically different sites that further vary in the underlying biological basis, histology and treatment response. Consequently, with treatment currently being administered based on clinical/histological parameters, accurate prognostication is still a rising challenge. Given the availability of multi-modality treatments as first line therapy, designing treatment strategies based on accurate understanding of treatment outcome is imperative; identification of molecular markers that can improve accuracy of prognosis is hence of extreme significance. The cataloging of biomarkers that correlate to tumor biology, its clinicopathological characteristics and evaluation of their prognostic ability is an essential step towards identifying candidate markers of clinical utility [8,24]. Significant advances in global profiling technologies has improved the understanding of the mechanisms underlying the disease and thereby generating a repertoire of biomarkers that can be assessed/validated for their clinical relevance. This study attempted to identify clinically relevant, molecular prognosticators pertaining to etiology, site, stage and pathological severity, by the meta-analysis approach.

Etiology based categorization was carried out in terms of HPV associated disease, HPV being the major cause of viral associated disease in HNSCC. Although oropharyngeal cancers are the major site with HPV-associated etiology, recent studies have attributed a role in oral cancers too [11] [25] [26]. In our study, although 63 genes, including RELB, the upregulation of which leads to TRAF regulated over expression of HPV E6 protein [27], only three showed a survival impact. RPPP25, NUDCD2 were poor prognosticators, while, interestingly, NOVA1 was a good prognosticator in patients with HPV. NOVA1 is known to be involved in the downregulation of E6 and E7 proteins in HPV associated cancers [28] and is hence indicated as a good prognosticator in HPV+ patients. These genes can be possible prognosticators in HPV+ cancers, subject to large scale clinical validation.

Site-specific meta-analysis of publicly available data identified a subset of genes that were common across the three major sites of HNSCC (tongue, laryngopharynx, oropharynx); the pathways that were primarily enriched included focal adhesion and proteoglycans in cancer, and cell cycle, mitotic pathway, which are well known in various cancers [29–37]. Differential expression profiling of the tongue cancer cohort triaged based on the stage, pathology identified multiple candidate markers; JAM2, SMURF1, LY6E, MFN1 and SUPT16H. JAM2 is reported to be instrumental for metastatic progression in breast and colon cancer [38,39], while others are known regulators of migratory/invasive properties in many cancers [31,40–44]. Analysis in laryngopharyngeal cohort also identified markers of early stage disease with PNI/ALI which included MFN1, PAK2, PIK3CA, FXR1 (differentials), PRRC2B and CBLL1 (with prognostic efficacy), all of which were previously identified to play a major role in head and neck oncogenesis as well as in other cancers [45–52] EPS8, identified in ECS- laryngopharyngeal patients in this study, is known to be involved in the proliferation apoptosis, adhesion and migration in other cancers including HNSCC [53–56]. The correlation of these markers with the clinically/pathologically classified sub-cohorts (ALI+/PNI+) indicated their possible relevance as predictive panel, with a subset of genes providing survival impact. A comparison of the prognosticators identified for tongue and laryngopharynx indicated that AP2M1 and IGF2BP2 are common across sites, with deregulation of AP2M1, a protein trafficking molecule, signifying contradictory effects in tongue and laryngeal cancer. NUDCD1 also known as CML66, located on the chromosome 8q23, was one of the significant unique poor prognosticator in advanced tongue cancers along with MTFR1, IGF2BP2, TSTA3; genes that associated with survival in tongue cancer, many of these genes have been identified as involved in tumorigenesis, metastases, immune suppression in solid tumors [57–60]. The prognosis of laryngeal cancers could be improved by addition of markers such as IL1RAP, LANCL2, RYK, and SLC33A1. IL1RAP, interleukin involved in synthesis of pro inflammatory proteins [61], RYK a member receptor protein tyrosine kinases have been reported in drug resistance, cell motility, anchorage independent cell growth and other tumorigenic properties [62–64].

In tongue and laryngopharyngeal cancers, advanced stage of the disease signifies poor prognosis. Nevertheless, studies have also shown a subset of early stage patients that have an extremely poor prognosis [65,66]. Biomarkers that can further classify these patients based on outcome can be immensely valuable in this context. Early stage tongue cancer patients were significantly classified into patients with poor/good survival based on alterations in AP2M1, CTBP1, and MTFR1 all of whom were associated with prognosis in other studies [60,67,68]. Additionally OTUD6B alterations, which was prognostic in advanced stage (III-IV) patients was also significant predictor of survival in early stage patients with perineural invasion/angiolymphatic invasion (Stage I-II/PNI+/ALI+), indicating that this marker is a clear indicator of advanced disease. Expectedly, the prognosticators of early stage laryngopharyngeal cohort were completely unique and included TWISTNB and UBE2Q1, previously designated poor prognosticators in breast and hepatocellular cancers [69,70].

Pathological parameters that include perineural invasion, angiolymphatic invasion, and extracapsular spread signify poor prognosis; assessment of markers associated with these parameters, identified the adjunct predictors. Interestingly, there were no common significant predictor of ALI/PNI across the two sites (MFN1 was a differential but did not show relevance as a prognosticator). In PNI/ALI+ tongue cancer, the panel of TFG (tumor suppressor gene) [71], TMEM267, BRIX1 (good prognosticators), NUDCD1 and PRKCI (poor prognosticators) together could categorize the patients based on survival; PRKC1 expression is known to be associated with metastasis and poor prognosis in esophageal/ovarian cancer [72,73]. The predictor panel in patients with PNI/ALI in laryngopharyngeal cancer consisted of AP2M1, LANCL2, PFN2, RPN1, MAP3K13, WWTR1 and IL1RAP; WWTR1 being a transcriptional coactivator regulating cell proliferation, differentiation, survival and apoptosis in oral cancer cells [74,75], while LANCL2 is associated with the EGFR pathway in many cancer [76,77]. The association of these markers with PNI and ALI indicated their possible role in the processes apart from their significance as adjunct prognosticators. Assessment of ECS, an additional prognosticator in advanced stage disease, indicated a distinct set of markers in tongue cancer (EXT1, GMPS, TSTA3), while in laryngopharyngeal cancers a majority of the markers were common those associated with late stage disease. Notably, in tongue cancer patients without ECS, the markers of advanced disease were poor prognosticators indicating their relevance as adjunct prognosticators in the absence of clinical/pathological parameters.

The oropharyngeal cohort was assessed separately, given the high prevalence of HPV-associated etiology. Low sample numbers precluded extensive validation in TCGA patient cohort; nevertheless a distinct alteration pattern of candidate genes was observed between HPV+ and HPV–cohorts of oropharyngeal cancers. A distinct set of prognosticators were identified for HPV+ and HPV- oropharyngeal cancers; ECHDC2, GGT6, HERC5 were showed to be associated with HPV+; HERC5 has a known anti-viral activity [78] [79] and its possible role in HNSCC HPV associated cancer is yet to be studied. On the other hand alterations in GRB10, EMILIN1, FNDC1, FOXF2 and HLX, determined poor prognosis in the HPV- cohort; this subset is involved in multiple carcinogenic processes in different solid tumors [80] [81] [82] [83] [84] [85].

The clinical relevance of molecular prognosticators is contentious, given the vast repertoire of data, and the lack of adequate validation to accurately pin-point the beneficial patient cohort. In view of the extremely clinical, pathological, cellular and molecular heterogeneity inherent in all cancers including head and neck cancers, the utility of the biomarkers need to be customized to the sub-categories of patients with definite clinico-pathological parameters prior to validation. This study was an attempt to leverage the existing high throughput studies to specify the predictive/prognostic marker pattern that correlate to the various clinic-pathological sub-types in tongue/ laryngopharyngeal/oropharyngeal cancers, the most common sub-sites of head and neck cancer. Although a common thread of pathways/biomarkers was observed, the distinct marker subset that represented each of the sub-cohorts emphasizes the point of discussion. The primary limitation was that this study was confined to three sub sites; it can be expanded to other sites in order to enable accurate triaging of patients based on risk and thereby provide appropriate treatment. Marker panels that classified the sub-cohorts with historically good prognosis, such as the patients with early stage disease, patients without extra-capsular spread, can prove to be invaluable candidates to improve on the current prognostic indicators (S2 Fig). Distinct and large scale clinical validation is mandatory prior to the adoption of these markers into a clinical setting; nevertheless this study points to the need of customized marker mapping in patients and provides a database of annotated candidates for subsequent validation.

## Supporting information

S1 Fig PRISMA flow diagram for selection of series for meta-analysis. The selection pipeline for the series is indicated including the comprehensive search criteria, screening process and details of the eligible studies.
(TIF)

S2 Fig Marker panel for prognostication of Head and neck cancer. A pictorial representation of all markers that were significantly associated with prognosticators of HPV associated cancer and different subsites of HNSCC (tongue, laryngopharyngeal and Oropharyngeal cancer) with various clinic-pathological parameters.
(TIF)

S1 File Details of microarray series used in the site specific meta-analysis. (XLSX)

S2 File List of concordant, differentially expressed genes obtained from tongue, larynx pharynx and oropharyngeal cancer. (XLSX)

S3 File Database annotation of significant gene entities from HPV associated cancer. List of differentially expressed genes obtained from HPV based analysis (A). List of Gene Ontology categories from TOPPFUN enrichment analysis for HPV associated cancer (B). List of KEGG and Reactome pathways obtained from TOPPFUN enrichment analysis for HPV associated cancer (C).
(XLSX)

S4 File List of significant gene entities which are differentially expressed in HPV associated cancer in TCGA. List of genes altered (gene expression) in >10% of the TCGA HPV specific cohort (A). List of genes altered (gene expression) in >10% of the TCGA HPV positive specific cohort (B). List of genes altered (gene expression) in >10% of the TCGA HPV negative specific cohort (C). List of significant genes altered (gene expression) only in HPV positive specific cohort (D). Kaplan-Meier analysis of individual genes for their survival in HPV positive cohort (E).
(XLSX)

S5 File Database annotation of significant gene entities and percentage alteration in patients with tongue cancer in TCGA. List of differentially expressed genes obtained from Tongue cancer (A). List of Gene Ontology categories from TOPPFUN enrichment analysis for tongue cancer gene entities (B). List of KEGG and Reactome pathways obtained from TOPPFUN enrichment analysis for tongue cancer gene entities (C). List of genes altered (gene expression) in >10% of the TCGA tongue specific cohort (D).
(XLSX)

S6 File List of significant gene entities which are differentially expressed and Kaplan-Meier curve analysis in various sub cohorts of patients with tongue cancer in TCGA. List of significant markers which are differentially expressed in various sub cohorts of patients with tongue cancer in TCGA (A). Kaplan-Meier curve analysis of significant gene entities in various sub cohorts of patient with tongue cancer in TCGA (B).
(XLSX)

S7 File Database annotation of significant gene entities and percentage alteration in patients with laryngopharyngeal cancer in TCGA. List of differentially expressed genes obtained from laryngopharyngeal specific cohort (A). Gene Ontology categories from TOPPFUN enrichment analysis for laryngopharyngeal cancer gene entities (B). KEGG and Reactome pathways obtained from TOPPFUN enrichment analysis for laryngopharyngeal cancer gene entities (C). List of genes altered (mRNA expression) in >10% of the TCGA laryngopharyngeal specific cohort (D).
(XLSX)

S8 File List of significant gene entities which are differentially expressed and Kaplan-Meier curve analysis in various sub cohorts of patients with laryngopharyngeal cancer in TCGA. List of significant gene entities which are differentially expressed in various sub cohorts of patients with laryngopharyngeal cancer in TCGA (A). Kaplan-Meier analysis of differential genes for their survival in various sub cohorts of patients with laryngopharyngeal cancer in TCGA (B).
(XLSX)

S9 File Kaplan-Meier curve analysis of significant gene entities in various sub cohorts of patient with laryngopharyngeal cancer in TCGA. Kaplan-Meier analysis of individual genes for their survival in TCGA laryngopharyngeal cancer cohort (A). Kaplan-Meier analysis of combination markers for their survival in TCGA laryngopharyngeal cancer cohort (B).
(XLSX)

S10 File Database annotation of significant gene entities and from oropharyngeal cancer. List of differentially expressed genes obtained from Oropharyngeal cohort (A). List of Gene Ontology categories from TOPPFUN enrichment analysis for Oropharyngeal cohort (B). List of KEGG and Reactome pathways obtained from TOPPFUN enrichment analysis for Oropharyngeal cohort (C).
(XLSX)

S11 File List of significant gene entities which are differentially expressed in oropharyngeal HPV positive and negative in TCGA. List of genes altered (gene expression) in >10% of the TCGA Oropharynx HPV specific cohort (A). List of genes altered (gene expression) in >10% of the TCGA Oropharynx HPV positive specific cohort (B). List of genes altered (gene expression) in >10% of the TCGA HPV negative specific cohort (C). List of genes altered (gene expression) only in HPV positive specific cohort (D).
(XLSX)

S12 File Kaplan-Meier curve analysis of significant gene entities in oropharyngeal HPV positive and negative in TCGA. Kaplan-Meier analysis of individual genes for their survival in Oropharyngeal HPV positive TCGA patients (A). Kaplan-Meier analysis of individual genes for their survival in Oropharyngeal HPV negative TCGA patients (B).
(XLSX)

S1 PRISMA Checklist (DOC)
