# Supplementary figures and images for: Molecular prognosticators in clinically and pathologically distinct cohorts of head and neck squamous cell carcinoma—A meta-analysis approach
Source: PLoS One. 2019 Jul 16;14(7):e0218989. doi: 10.1371/journal.pone.0218989 (PMC6634788; doi:10.1371/journal.pone.0218989)

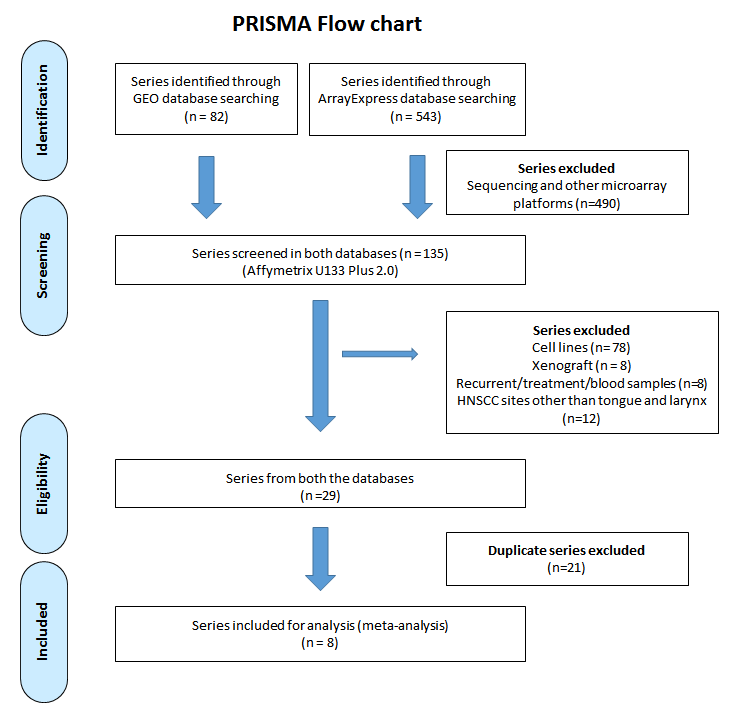

Supplement: S1 Fig — The selection pipeline for the series is indicated including the comprehensive search criteria, screening process and details of the eligible studies. (TIF) [file pone.0218989.s001.tif]

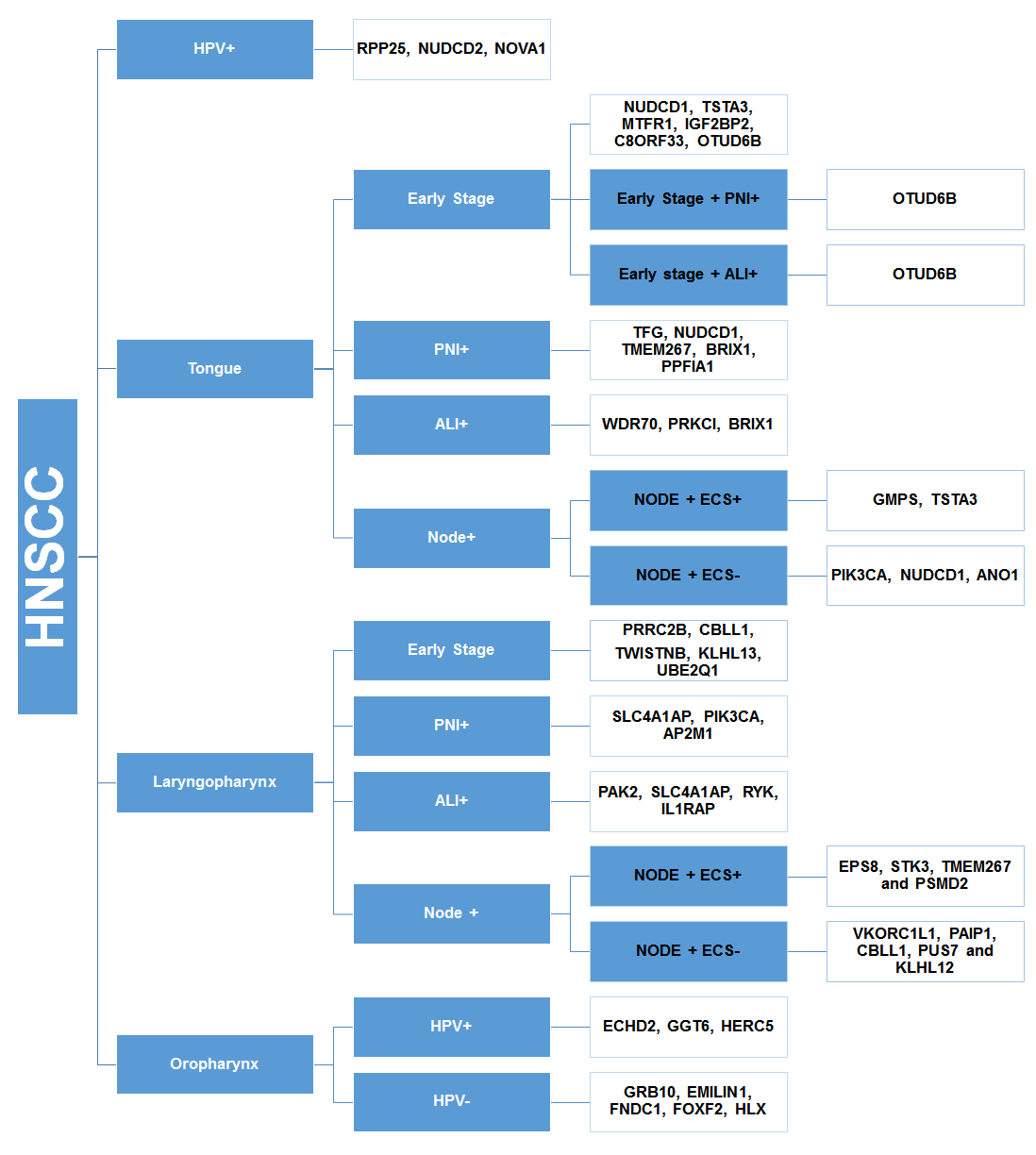

Supplement: S2 Fig — A pictorial representation of all markers that were significantly associated with prognosticators of HPV associated cancer and different subsites of HNSCC (tongue, laryngopharyngeal and Oropharyngeal cancer) with various clinic-pathological parameters. (TIF) [file pone.0218989.s002.tif]
